# The key genes and pathways related to male sterility of eggplant revealed by comparative transcriptome analysis

**DOI:** 10.1186/s12870-018-1430-2

**Published:** 2018-09-24

**Authors:** Yan Yang, Shengyou Bao, Xiaohui Zhou, Jun Liu, Yong Zhuang

**Affiliations:** 1 College of Horticulture, Nanjing Agricultural University, Nanjing, 210095 China; 2 Institute of Vegetable Crops, Jiangsu Academy of Agricultural Sciences, Nanjing, 210014 China; 3 Jiangsu Key Laboratory for Horticultural Crop Genetic Improvement, Nanjing, 210014 China

**Keywords:** Eggplant, Male sterility, RNA-seq, DEGs

## Abstract

**Background:**

Male sterility (MS) is an effective tool for hybrid production. Although MS has been widely reported in other plants, such as Arabidopsis and rice, the molecular mechanism of MS in eggplant is largely unknown. To understand the mechanism, the comparative transcriptomic file of MS line and its maintainer line was analyzed with the RNA-seq technology.

**Results:**

A total of 11,7695 unigenes were assembled and 19,652 differentially expressed genes (DEGs) were obtained. The results showed that 1,716 DEGs were shared in the three stages. Gene ontology (GO) and Kyoto Encyclopedia of Genes and Genomes (KEGG) analysis indicated that these DEGs were mainly involved in oxidation-reduction, carbohydrate and amino acid metabolism. Moreover, transcriptional regulation was also the impact effector for MS and anther development. Weighted correlation network analysis (WGCNA) showed two modules might be responsible for MS, which was similar to hierarchical cluster analysis.

**Conclusions:**

A number of genes and pathways associated with MS were found in this study. This study threw light on the molecular mechanism of MS and identified several key genes related to MS in eggplant.

**Electronic supplementary material:**

The online version of this article (10.1186/s12870-018-1430-2) contains supplementary material, which is available to authorized users.

## Background

Male sterility (MS) exits widely in the flowering plants. It can be used as a tool for breeders to create new hybrid varieties. The hybrids present heterotic vigor, high uniformity, adaption, tolerance and yield. Moreover, hybrids are protected against unauthorized multiplication [1]. Therefore, MS is of interest due to its application in hybrid seed production [2, 3]. The discovery and utilization of MS plants can provide excellent germplasm resources for yields increase, quality improvement and resistance enhancement, which have a practical value in eggplant breeding. The study of the molecular mechanism of MS provides a crucial guiding significance for breeding programs.

The development of the anther is a complex process involving numerous pathways. The molecular mechanisms of anther development and MS have been reported in previous studies [4–7]. A large number of genes are related to anther development, including genes encoding several proteins in energy transduction [8–10], lipid transport and metabolism [11, 12], carbohydrate metabolism [13–15]. The ATPase subunit 6 (*atp6*) is an important mitochondrial functional gene involved in energy supply. The 33-bp insert and 3-bp deletion of this gene were characterized specific to cytoplasmic male sterility based on the analysis of 104 kenaf varieties [16]. For lipid transfer, *CaMF2*, encoding a lipid transfer protein, was identified as a functional gene in pollen development as virus-induced gene silencing of *CaMF2* affected pollen development in *Capsicum annuum* L. This indicated that lipid transfer was a key effector for male fertility [17]. In Datta’s [18] study, significant differences in the level of carbohydrates, specifically, hexose sugars and the carbohydrate genes expression were observed between male fertile and sterile lines.

Transcription factors play a vital role in plant development [19–21]. A large number of studies have shown them to be pivotal in anther or pollen development [22, 23]. *SPOROCYTELESS (SPL)/NOZZLE (NZZ)*, encoding a MADS box transcription factor, was required for the development of anther. *SPL* gene is activated by the floral homeotic gene *AG* in the archesporial cell. The SPL protein recruited TPL/TPR using its EAR motif to promote sporogenesis by suppressing target genes of TCP transcription factors [24]. These studies suggested that there exists a complex cascade regulation during anther development [25]. A large number of transcription factor families have been also identified in tapetum and participate in the development of anthers. For example, the mutant DYSFUNCTIONAL TAPETUM1 (DYT1), a member of the bHLH transcription factor family, affected the expression levels of many tapetum-related genes and the mutant exhibits an abnormal anther phenotype [26]. ABORTED MICROSPORE (AMS) interacted with two other bHLH proteins to regulate the expression of genes which were related to tapetal cell developments [27, 28].

A number of genes involved in MS have been defined and the network has been acknowledged as complicated [29–31]. With the increasing popularity of eggplant all over the world, the utilization of MS lines in eggplant breeding is more and more important to produce hybrid seeds. Although the molecular mechanism of MS has been investigated in several crops and model plants, it remains poorly understood in the eggplant. Researchers have long known that genetic MS is caused by recessive nuclear genes [3, 32]. MS was induced by suppressing transcription factors and fertility could be reversed by ethanol [33, 34]. Furthermore, the differences in the DNA sequences flanking of five mitochondrial *ATP* and *COX* genes were verified to cause cytoplasmic male sterility (CMS) in eggplant and the report showed novel open reading frames (*orfs*) were causal genes for each type of CMS [35]. In this study, we have used the broadly-used RNA-seq technology to undertake a comparative transcriptome analysis of the male sterile line and its maintainer line to identify the MS-related genes and pathways in the eggplant.

## Results

### The phenotype of eggplant anthers

**Fig. 1:**
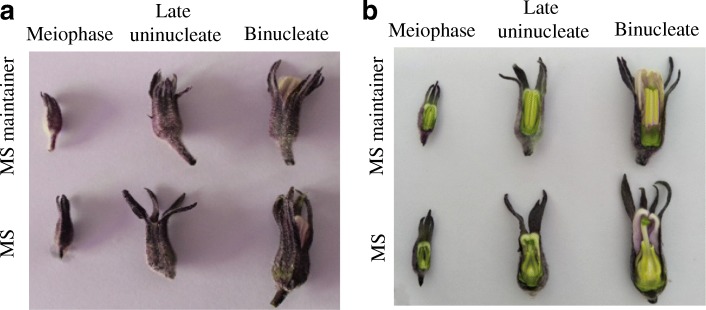
The morphological characteristics of flower buds in different stages. **a** The morphology of flower buds. **b** The morphology of anthers

In this study, a MS line and its maintainer line were chosen to perform a comparative transcriptional analysis. The flower buds were extracted from this two lines at three different developmental stages. As shown in Fig. 1a, the appearance of buds between the MS and the maintainer lines showed no significant difference. However, the anthers of MS line were greenish, flat and loose arranged while the anthers of the maintainer tended to be yellow, full and more cohesive (Fig. 1b). Significantly, the stigma of the maintainer line almost was enveloped by anthers while the stigma of the MS line was not (Fig. 1b).

### Identification of differentially expressed genes

**Fig. 2:**
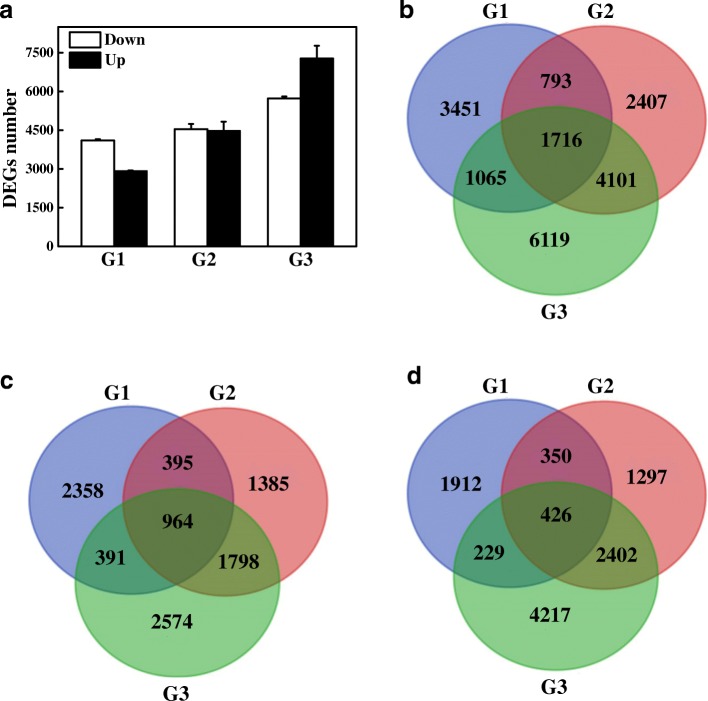
The number of DEGs between the maintainer line and MS line at meiophase, late uninucleate and binucleate stages. **a** The total number of up-regulated and down-regulated DEGs. **b** Venn diagram of all DEGs. **c** Venn diagram of up-regulated genes. **d** Venn diagram of down-regulated genes

To characterize DEGs in the MS line, we first preformed RNA-seq on six samples with three repeats using the Illumina Seq platform. As a result, a total of 414 million clean reads were obtained from the samples. The reads were assembled into contigs and 11,7695 unigenes. This was much more than the predicted genes in the draft genome in which eggplant has 85,446 genes [36]. The major reasons for this were as follows: Firstly, one gene can be transcribed into two transcripts. If the two transcripts differ significantly, the assembly software will identify them as two genes. Secondly, de novo assembly cannot be completed with the repeated sequence. As a result, one gene will be identified as multiple genes. Using the FPKM method (Fragments PerKilo base of transcript per Million mapped reads) to calculate the expression level of the unigenes and DESeq2 to filter these unigenes, 7,025 (4,108 up-regulated, 2,917 down-regulated), 9,017 (4542 up-regulated, 4,475 down-regulated) and 13,001 (5,727 up-regulated, 7,274 down-regulated) DEGs were found in the MS line at three stages, respectively (Fig. 2a). In total, 19,652 DEGs were obtained and of these, 1,716 (964 commonly up-regulated and 426 commonly down-regulated) genes were shared among three stages (Fig. 2b, c, d, Additional file 1: Table S1).

### Hierarchical clustering analysis

**Fig. 3:**
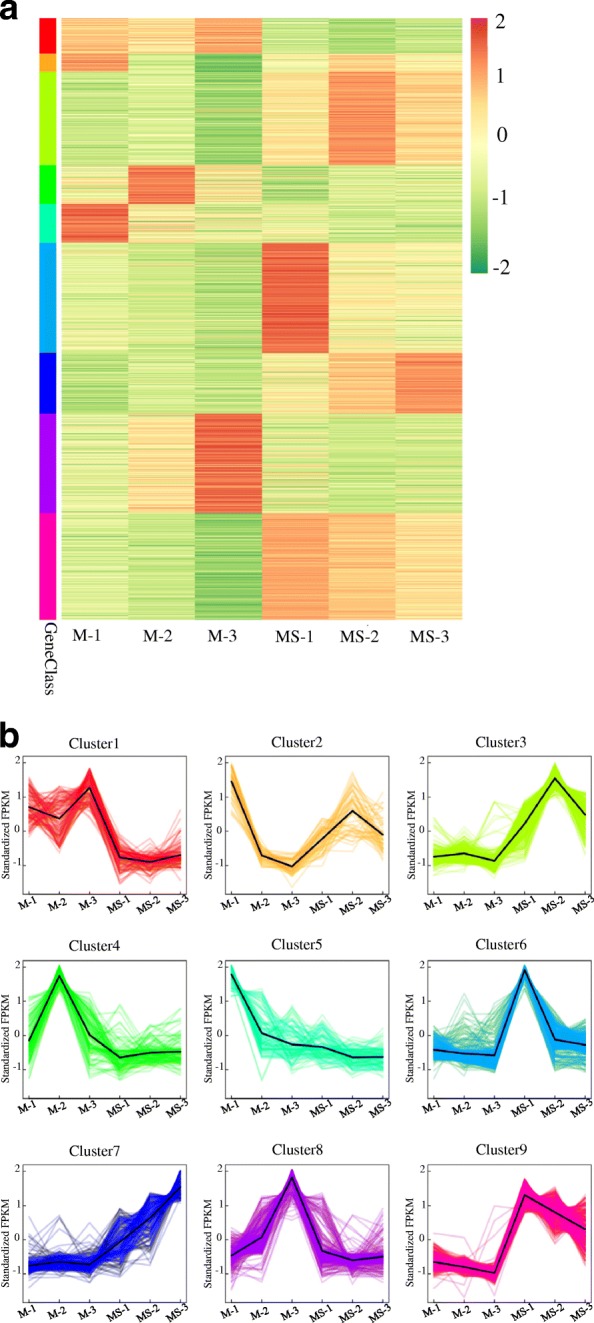
Clustering analysis of 1,716 DEGs between the maintainer line and the MS line. **a** Hierarchical clustering of the 1,716 DEGs. **b** Expression patterns of the 1,716 DEGs in the nine clusters. M-1, M-2 and M-3 represent the maintainer line at meiophase, late uninucleate and binucleate stage, respectively. MS-1, MS-2 and MS-3 represent the MS line at meiophase, late uninucleate and binucleate stage, respectively

Based on the similarity of gene expression, hierarchical clustering was performed on the 1,716 DEGs among those samples (Fig. 3). Hierarchical clustering of the genes expression profiles of the MS line and maintainer line with different stages showed that the expression pattern was different among these samples. The DEGs were categorized into nine gene clusters (Fig. 3, Additional file 2: Table S2). Cluster 1, cluster 4 and cluster 5 showed a relatively high transcript level in the maintainer line. These three clusters mainly included genes encoding proteins related to energy production and conversion (TRINITY_DN21187_c0_g1, TRINITY_DN21187_c0_g2 and TRINITY_DN82756_c2_g5), amino acid transport and metabolism (TRINITY_DN21153_c0_g1, TRINITY_DN166456_c0_g1, TRINITY_DN169613_c0_g1 and TRINITY_DN38463_c0_g1) and carbohydrate transport and metabolism (TRINITY_DN56811_c0_g2, TRINITY_DN63174_c0_g13 TRINITY_DN85580_c0_g1 and TRINITY_DN67079_c0_g2). The expression of the above genes was inhibited in the MS line. The result indicated that these genes may be related to MS. However, some genes in cluster 3, cluster 7 and cluster 9 expressed at a lower level in the maintainer line compared with the MS line. For example, genes mainly in posttranslational modification, protein turnover and encoding chaperones (TRINITY_DN34505_c0_g1, TRINITY_DN63937_c0_g2 and TRINITY_DN5769_c0_g3) and transcription factors (TRINITY_DN85424_c0_g1, TRINITY_DN82628_c0_g1 and TRINITY_DN84016_c2_g4) were highly expressed in the MS line. In addition, signal transduction genes including the protein kinases (TRINITY_DN22944_c0_g1, TRINITY_DN77773_c1_g2 and TRINITY_DN134628_c0_g1) were also up-regulated in the MS line. The rest of DEGs were clustered into cluster 2, cluster 6 and cluster 8 based on their expression patterns.

GO enrichment analysis of these six clusters which may be relevant to MS was performed subsequently. The GO terms related to energy production and conversion, carbohydrate transport and metabolism and amino acid transport and metabolism were highly enriched in cluster 1,cluster 4 and cluster5, such as GO:0016174 (NAD(P)H oxidase activity), GO:0048040 (UDP-glucuronate decarboxylase activity), GO:0035999 (tetrahydrofolate interconversion), GO:0034219 (carbohydrate transmembrane transport), GO:0051119 (sugar transmembrane transporter activity), GO:0004794 (L-threonine ammonia-lyase activity) and GO:0009097(isoleucine biosynthetic process) etc. (Additional file 3: Figure S1, Additional file 4: Figure S2 and Additional file 5: Figure S3). It is of interest that the transcription factor (GO:0003700) and regulation of transcription (GO:0006355) were the most enriched terms of cluster 3, cluster 7 and cluster 9 in which the transcript level of genes in MS line was higher. In addition, the GO terms associated with inorganic ion transport and metabolism included GO:0046916 (cellular transition metal ion homeostasis), GO:0046914 (transition metal ion binding) and GO:0030001 (metal ion transport) (Additional file 6: Figure S4, Additional file 7: Figure S5 and Additional file 8: Figure S6).

### Gene ontology and pathway analysis of DEGs

**Fig. 4:**
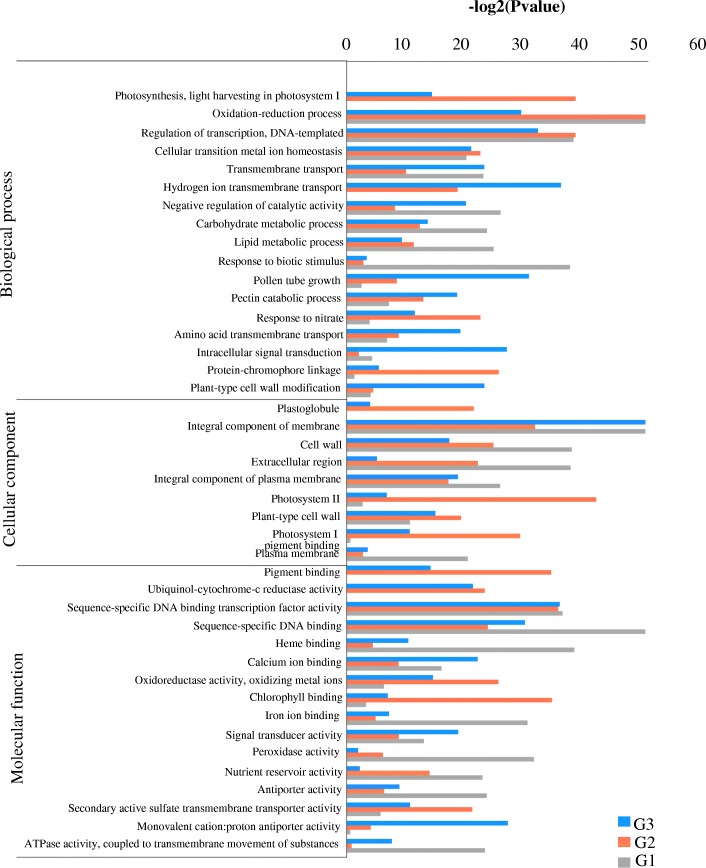
Analysis of GO enrichment for DEGs at the three stages of bud development. G1, G2 and G3 represent the DEGs between the maintainer line and MS line at meiophase, lateuninucleate stage and binucleate stage, respectively

**Fig. 5:**
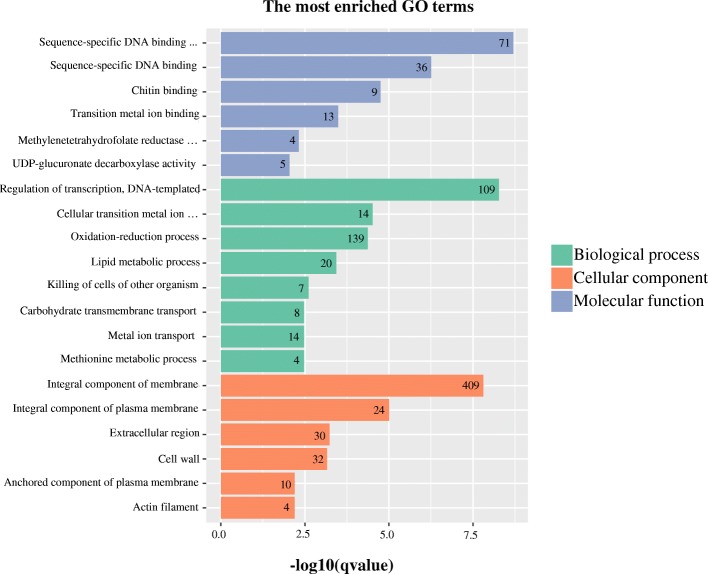
Analysis of GO enrichment for 1,716 common DEGs

GO enrichment analysis was used to promote the global analysis of DEGs. The DEGs were classified into three categories of ontologies including “biological process” (BP), “molecular function” (MF) and “cellular component” (CC). Among the BPs, these DEGs in three stages were mainly involved in oxidation-reduction process (GO:0055114), regulation of transcription and DNA-templated (GO:0006355), cellular transition metal ion homeostasis (GO:0046916), lipid metabolic process (GO:0006629) and carbohydrate metabolic process (GO:0005975) (Fig. 4). The MFs contained 498, 639 and 780 GO terms in G1, G2 and G3, respectively. Among these terms, GO:0003700 (sequence-specific DNA binding transcription factor activity), GO:0043565 (sequence-specific DNA binding) and GO:0005509 (calcium ion binding) were highly enriched in all the stages (Fig. 4). Furthermore, the highly enriched terms included in CC among three stages were GO:0016021 (integral component of membrane), GO:0005618 (cell wall) and GO:0005887 (integral component of plasma membrane) (Fig. 4). The 1,716 shared DEGs enriched in regulation of transcription, sequence-specific DNA binding transcription factor activity and integral component of membrane (Fig. 5). These results indicated that the anther development in the MS line may be related to different genes.

**Fig. 6:**
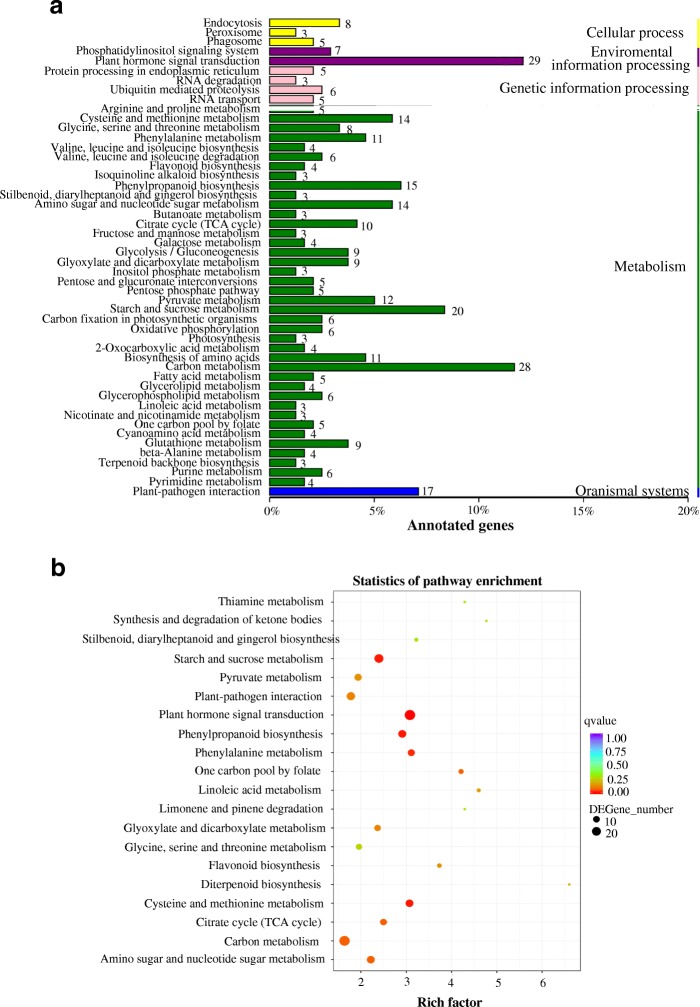
KEGG pathway enrichment analysis. **a** Statistic analysis of annotated genes in KEGG pathways. **b** Scatterplot of KEGG pathway enrichment for DEGs

Pathway enrichment analysis was usually employed to identify the major biochemical and signal transduction pathways in which the DEGs were involved. Accordingly, we explored the biological functions of these DEGs by pathway enrichment analysis. A total of 107, 115 and 120 DEGs were significantly annotated in the three stages by KEGG pathway, respectively, while 76 pathway categories were enriched in the 1,716 shared DEGs (Additional file 9: Table S3). As shown in Fig. 6, plant hormone signal transduction (ko04075), carbon metabolism (ko01200) and starch and sucrose metabolism (ko00500) were the three most significantly entries. Interestingly, some components overlapped in different pathways. These results showed that multiple pathways may contribute to anther development within a complicated pathway network.

### Identification of differentially expressed transcription factors

**Fig. 7:**
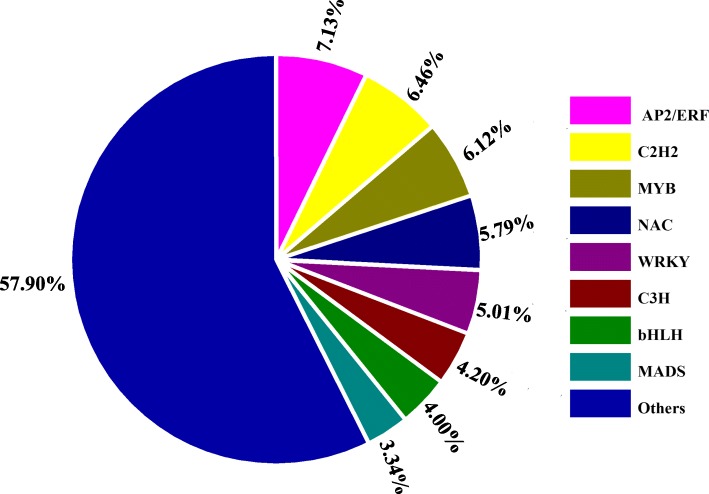
Distribution of differentially expressed transcription factors

A large number of studies about MS have shown that a high percentage of sterility-associated genes in Arabidopsis encode transcription factors (TFs) [37, 38]. In previous analysis, we have found some DEGs belonged to genes encoding TFs. To better understand the molecular mechanism of MS, we analyzed the differentially expressed TFs in this study. 898 TFs (498 commonly up-regulated and 216 commonly down-regulated) shared in three stages were totally identified from the DEGs (Additional file 1: Table S1). These TFs could be classified into 63 families, among which the main ones were AP2/ERF family (7.13%), C2H2 family (6.46%), MYB family (6.12%), NAC family (5.79%), WRKY family (5.01%), C3H family (4.2%), bHLH family (4.00%) and the MADS family (3.34%) (Fig. 7). Interestingly, although some unigenes were annotated to the same kind of transcription factor, such as the predicted NAC- encoded genes TRINITY_DN70641_c0_g1 (up-regulated) and TRINITY_DN73046_c0_g1 (down-regulated), their expression patterns were different between the MS and maintainer line (Additional file 1: Table S1). These results suggested that it is an extremely intricate and complex transcriptional network for anther development.

### Correlation network analysis with WGCNA

**Fig. 8:**
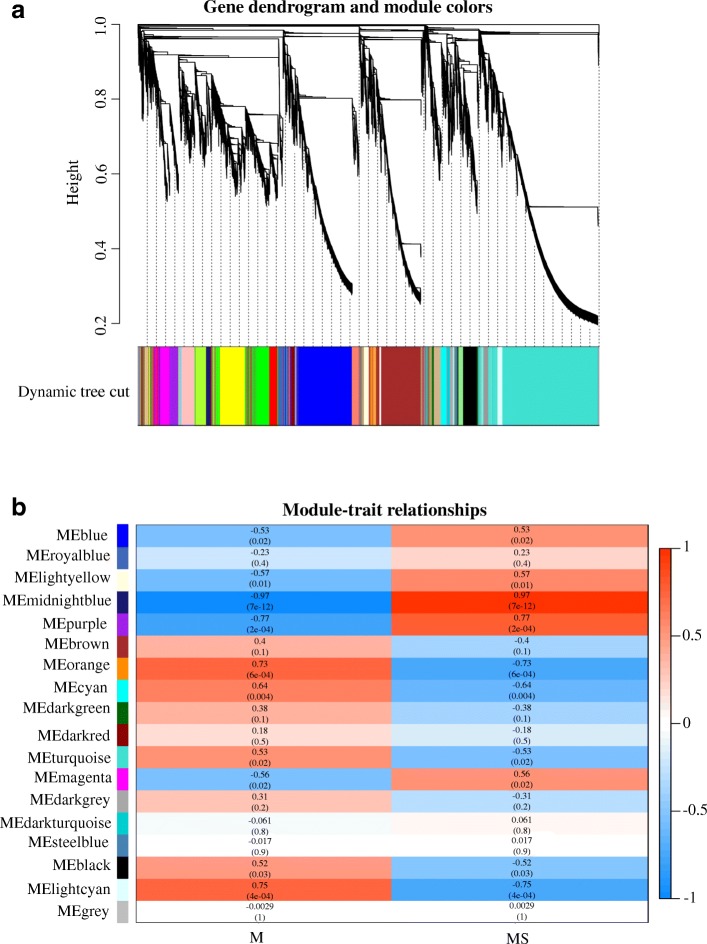
Weighted correlation network analysis of MS-related genes. **a** Heriarchical clustering tree showing co-expresssion modules. Each leaf in the tree is one gene. The major tree branches constitute 17 modules labeled by different colors. **b** Module-trait relationship. The left lane indicates 17 module eigengenes. The right lane indicates the module-trait correlation from − 1 to 1. M represents the maintainer line and MS represents the male sterile line

WGCNA is an alternative tool to analyze the target genes at a network-level [39, 40]. To obtain the genes correlated with MS, weighted correlation network was constructed with the differentially expressed genes. In this study, 17 modules were identified from the RNA-seq data (Fig. 8a). The module eigengenes for 17 modules were correlated with different samples. Analysis of the module-trait relationship showed that the module ‘lightcyan’ (*r* = 0.75, *p* = 4e-04) and ‘midnightblue’ (*r* = 0.97, *p* = 7e-12) were highly correlated with MS (Fig. 8b). The module ‘lightcyan’ included 539 genes mainly containing genes related to carbohydrate, amino acid and lipid metabolism (Additional file 10: Table S4), while 1,174 genes mainly including genes encoding transcription factors or correlated to signal transduction were identified in the module ‘midnightblue’ (Additional file 11: Table S5). For example, a protein NRT1/ PTR (TRINITY_DN57038_c1_g1) in the module ‘lightcyan’ and a Myb-related protein (TRINITY_DN82628_c0_g1) in the module ‘midnightblue’ were differentially expressed. These results were almost consistent with earlier hierarchical clustering analysis.

To further understand the mechanism of MS, the GO enrichment and KEGG pathway of unigenes in the two modules were analyzed. The unigenes in “lightcyan” module were mainly enriched in assembly of proteasome core complex (GO:0080129), photorespiration (GO:0009853), mitochondrial respiratory chain complex I (GO:0005747) and NADH dehydrogenase (ubiquinone) activity (GO:0008137) (Additional file 12: Figure S7), while the highly enriched terms of the “midnightblue” module were associated with regulation of transcription and DNA-templated (GO:0006355), integral component of membrane (GO:0016021) and sequence-specific DNA binding transcription factor activity (GO:0003700) (Additional file 13: Figure S8). As shown in Fig. S7, the top 20 enriched terms of “lightcyan” module were related to respiratory and energy production. The most significantly entries in KEGG analysis of the “lightcyan” and “midnightblue” module were oxidative phosphorylation (ko00190) and plant hormone signaling system, respectively (Additional file 14: Figure S9, Additional file 15: Figure S10).

### Validation of differentially expressed genes

**Fig. 9:**
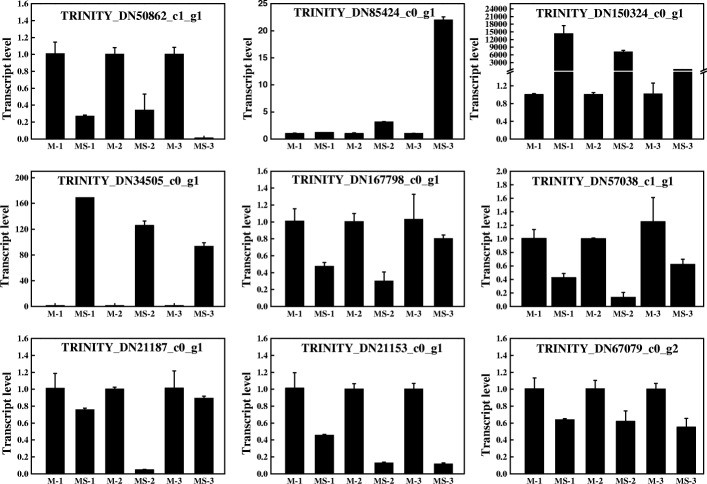
Verification of the expression of selected DEGs by qRT-PCR. M-1, M-2 and M-3 represent the maintainer line at meiophase, late uninucleate and binucleate stage, respectively. MS-1, MS-2 and MS-3 represent the MS line at meiophase, late uninucleate and binucleate stage, respectively

To verify the RNA-seq data, qRT-PCR was conducted on nine selected genes. According to the RNA-seq data, the selected genes were differentially expressed. As shown in the Fig. 9, the transcript level of the genes mentioned earlier was lower in the MS line, compared with the maintainer line. For example, amino acid-related methylenetetrahydrofolate reductase (TRINITY_DN21153_c0_g1)**,** fatty acid hydroxylase (TRINITY_DN50862_c1_g1), malate dehydrogenase (TRINITY_DN21187_c0_g1) and glucose-6-phosphate 1-dehydrogenase (TRINITY_DN67079_c0_g2) were suppressed in the MS line, while a gene related to posttranslation modification and chaperones (TRINITY_DN34505_c0_g1) and a NAC transcription factor (TRINITY_DN85424_c0_g1) were reduced in the maintainer line (Fig. 9). Most of these genes were mentioned earlier. Likewise, two unrelated genes (TRINITY_DN167798_c0_g1 and TRINITY_DN150324_c0_g1) were expressed at a lower and higher level in the MS line, respectively (Fig. 9), which was consistent with the transcriptome profile. Moreover, NRT1/ PTR (nitrate transporter/ peptide transporter) family was suppressed in the MS line, such as TRINITY_DN57038_c1_g1 in the module ‘midnightblue’ (Fig. 8). The consistency between RNA-seq data and qRT-PCR data manifested that the RNA-seq is highly reliable.

## Discussion

### The genes involved in respiratory and energy production are related to MS

The DEGs related to energy production and conversion were clustered and expressed higher in the maintainer line (Additional file 12: Figure S7). GO terms analysis showed that a large number of DEGs enriched in “oxidative-reduction” of BP and “oxidoreductase activity, oxidizing metal ions” of MF (Fig. 4). As a result, these drastically down-regulated genes functioned in energy production and conversion included NADH-related dehydrogenase (TRINITY_DN82756_c2_g5), ADP/ATP carrier protein (TRINITY_DN101445_c0_g1) and oxidoreductase (TRINITY_DN71824_c0_g2). These proteins are components in the mitochondrial respiratory chain. It suggests that mitochondrial respiratory related enzymes play a vital role in the eggplant MS line. The result is consistent with a previous study [41], in which the CMS related genes was explored in MS line of welsh onion. As we know, MS of many plants were associated with mitochondria, especially the CMS. Several researches have reported that the genes encoding mitochondrial respiratory chain enzymes and enzyme complexes were important to CMS lines in other plants [42, 43]. Therefore, our results are consistence with previous studies indicating the reduction in mitochondrial respiratory results in MS in eggplant.

### The genes involved in carbohydrate metabolic pathways are related to MS

The carbohydrate metabolism pathway is one of the basic metabolic pathway during plant development. It supplies energy and carbohydrates for plants growth and development [44]. Glycosyltransferase (TRINITY_DN105743_c0_g1) and glycosyl hydrolases (TRINITY_DN56811_c0_g2) belonging to two types of enzymes in the carbohydrate metabolism pathway were down-regulated in the MS line in our study. The genes encoding the two enzyme families have been reported to be associated with cell-wall synthesis and degradation [45–47]. SPG2, a GT43 glycosyltansferase and UPEX1, a GT31 glycosyltransferase were involved in the formation of pollen wall primexine [48, 49]. These results suggest that glycosyltransferase and glycosyl hydrolases play a specific role in pollen development. In this study, we found the most enriched terms were carbon, starch and sucrose metabolism in KEGG analysis and the genes related to carbohydrate transport were clustered together in hierarchical clustering analysis, which were consistent with the conclusions from other studies. Interestingly, few of microspores were observed in our MS line. Therefore, we speculate that the decrease in carbohydrate metabolism influences pollen formation, leading to MS in eggplant.

### The genes involved in amino acid transport and metabolic pathway are related to MS

Previously, it has been reported that glutamine in plant amino acid metabolism plays a central role during pollen development. The pollen population did not mature with glutamine starvation [50–52]. Fang [53] found that the expression of glutamine synthetase in the amino acid synthesis pathway decreased in pepper CMS. In accord with previous studies, one of the dramatically down-regulated genes in the MS line encoded glutamine synthetase (GS) (TRINITY_DN8287_c0_g1) in the study, indicating that the MS may result from the lack of glutamine synthetase. Another enzyme involved in amino acid metabolism in this study was NRT1/ PTR (nitrate transporter/ peptide transporter) family (TRINITY_DN134037_c0_g2, TRINITY_DN66882_c0_g2 and TRINITY_DN57038_c1_g1). Similarly, the abundance of NRT/PTR proteins was observed to decrease in Weichert’s [54] study. Researchers discovered that AtPTR5 mediated peptide transport into pollen through mutation and overexpression [55]. Hence, we infer that the MS in eggplant may be generated from a suppressed expression of NRT/PTR or other proteins that reduce transportation of peptides to affect pollen development.

### Transcription factors regulated the MS related genes

A large number of studies have established that TFs regulate their targets by binding to the cis-elements in the promoters through an interaction with protein partners during plant growth and development and response to environmental stimuli. In the MS line, the expression of some TFs was also altered. 898 differentially expressed TFs could be classified into 63 families including AP2/ERF, MYB, NAC, WRKY, bHLH and MADS (Fig. 7). Transcriptional regulation has been demonstrated to be important for male fertility. A bHLH transcription factor, DYT1 can activate the expression of two TFs- *MYB35* and *MS1*, which influences the tapetum function and pollen development [56]. With further investigation, it was found that *DYT1* directly regulates the expression of *TDF1* (*DEFECTIVE in TAPETAL DEVELOPMENT and FUNCTION1,* a putative R2R3 MYB transcription factor) [57], which in turn promotes the expression of *AMS* that is a regulator of pollen wall formation [58]. *AMS* acts upstream of *MS188* that affects the expression of *MS1* [58]. These results show that these transcription factors form a genetic pathway in pollen development. Our study has found more TFs than previous studies, suggesting that it is a more complicated transcriptional regulation network operative in pollen development in eggplant. Some TFs belonging to the same family were up-regulated and other were down-regulated, which complies with a recent study of bud dormancy in grapevine [59] (Additional file 1: Table S1). The result demonstrated that the members from a same TF family may play different roles in pollen development, which may constitute a more complex transcriptional regulatory network.

## Conclusions

In this study, a comparative transcriptome analysis was conducted between the male sterile line EP26A and its maintainer line EP26. In total, 19,652 DEGs were obtained. 1,716 genes were shared among three stages of pollen development. Hierarchical clustering, GO, KEGG analysis of the DEGs showed that these DEGs were mainly involved in oxidation-reduction, carbohydrate and amino acid metabolism and transcription regulations, which indicated that the genes and pathways may be related to MS. WGCNA revealed two modules significantly associated with MS. Therefore, our study elucidated the key genes and pathways related to MS of eggplant, which provided a theoretical basis and foundation for further research on fertility and anther development in eggplant.

## Methods

### Plant materials

In this study, a male sterile line EP26A and its maintainer line EP26 were employed. The maintainer line was an advanced-generation inbred line. The male sterile line was obtained from the continuously backcross of male sterile plant in progenies F2 of interspecific hybrid (*Solanum aethiopicum* × *Solanum melongena*) [60]. To eliminate the background, flower buds at meiophase (G1), lateuninucleate stage (G2) and binucleate stage (G3) were collected respectively. The anthers were extracted from flower buds, frozen immediately in liquid nitrogen and stored at − 80 °C. Every sample had three biological replicates that were sequenced independently.

### RNA extraction, library preparation, sequencing and transcriptome assembly

Total RNA of each sample was extracted using TRIZOL kit according the manufacture’s protocol. RNA qualification was monitored on 1% agarose gels. The integrity and purity of total RNA were checked using Qubit® RNA Assay Kit in Qubit® 2.0 Flurometer (Life Technologies, CA, USA) and the NanoPhotometer® spectrophotometer (IMPLEN, CA, USA), respectively. Sequencing libraries were generated using NEBNext® Ultra™RNA Library Prep Kit for Illumina® (NEB, USA) following manufacturer’s recommendations and index codes were added to attribute sequences to each sample. The Agilent Bioanalyzer 2100 system was applied to assess the library quality. The library preparations were sequenced on an Illumina platform and paired-end reads were generated, following the clustering of the index-coded sample performed on a cBot Cluster Generation System using TruSeq PE Cluster Kit v3-cBot-HS (Illumina). Clean reads were obtained by removing reads containing adapter, reads containing ploy-N and low quality reads from raw data. Due to the relatively poor assembly of eggplant genome, the self-assembly was used in this study [61]. Transcriptome assembly was accomplished using Trinity v2.2.0 [62]. The IDs of unigenes were automatically generated by the software subsequently. The read counts of unigenes were calculated by the software RSEM v1.2.19.

### Gene functional annotation

The function of all the unigenes were annotated based on the protein databases, including NCBI non-redundant database, Swiss-Prot, Cluster of Orthologous Groups (COG) and KEGG database. DESeq2 was used to filter DEGs with a fold change≥2 and a threshold of false discovery rates (FDR < 0.01). GO enrichment analysis of the DEGs was implemented by the GO seq R packages based Wallenius non-centreal hyper-genometric distribution. For pathway enrichment analysis, KOBAS 2.0 (https://www.biostars.org/p/200126/) was employed and a threshold of FDR ≤ 0.05 was defined. For identification of the transcription factor (TF), iTAK software was used to predict the TFs and PlnTFDB and PlantTFDB were used as the reference TF database. Putative TFs in eggplant were identified using BLASTx.

### Weighted correlation network analysis of male sterile-related genes

All the differentially expressed genes were used to build a correlation network using the WGCNA R package [63]. The adjacency matrix was generated by calculating the Pearson’s correlations among all genes. The power β was chosen based on the scale-free topology criterion [63]. The topological overlap measure (TOM) was calculated using the adjacency matrix. The dissimilarity TOM was used to construct the dendrogram. The modules were detected as branches of the dendrogram using the dynamic tree-cut [64] and a cut-off height of 0.25 was used to merge the branches to final modules. The modules were colored. The module eigengene (ME) value was calculated and used to estimate the association of modules with MS.

### Validation of gene expression

To validate the RNA-Seq data, the transcript levels of selected up- or down-regulated genes were confirmed by quantitative reverse transcription-polymerase chain reaction (qRT-PCR). Specific primers were designed in the website (http://primer3.ut.ee/) (Additional file 16: Table S6). RNA was reverse transcribed using the ReverTran Ace® qPCR RT kit (Toyobo). qRT-PCR was performed on an LightCycler 480 II (Roche, Switzerland) using the SYBR® Green qPCR Master Mixes (Takara, Japan). The PCR conditions consisted of denaturation at 95 °C for 30s, followed by 40 cycles of denaturation at 95 °C for 5 s, annealing and extension at 60 °C for 30 s. Melt curve analysis was performed to determine the specificity of reactions. The relative expression levels were calculated according to the ΔΔCt method. The eggplant Actin gene (Sme2.5_01462.1_g00018.1) was used as the internal control.

## Additional files

Additional file 1: **Table S1.** The differentially expressed genes between the MS line and its maintainer line. (XLSX 4983 kb)

Additional file 2: **Table S2.** The expression patterns of 1,716 shared DEGs. (XLSX 461 kb)

Additional file 3: **Figure S1.** Analysis of GO enrichment for genes in cluster1. (PPTX 67 kb)

Additional file 4: **Figure S2.** Analysis of GO enrichment for genes in cluster4. (PPTX 66 kb)

Additional file 5: **Figure S3.** Analysis of GO enrichment for genes in cluster5. (PPTX 66 kb)

Additional file 6: **Figure S4.** Analysis of GO enrichment for genes in cluster3. (PPTX 67 kb)

Additional file 7: **Figure S5.** Analysis of GO enrichment for genes in cluster7. (PPTX 66 kb)

Additional file 8: **Figure S6.** Analysis of GO enrichment for genes in cluster9. (PPTX 64 kb)

Additional file 9: **Table S3.** KEGG enrichment analysis for DEGs at three stages and 1,716 shared DEGs. (XLSX 114 kb)

Additional file 10: **Table S4.** The genes in module “lightcyan”. (XLSX 173 kb)

Additional file 11: **Table S5.** The genes in module “midnightblue”. (XLSX 323 kb)

Additional file 12: **Figure S7.** Analysis of GO enrichment for genes in “lightcyan” module. (PPTX 128 kb)

Additional file 13: **Figure S8.** Analysis of GO enrichment for genes in “midnightblue” module. (PPTX 83 kb)

Additional file 14: **Figure S9.** KEGG enrichment analysis for genes in “lightcyan” module. **a** Statistic analysis of annotated genes in KEGG pathways. **b** Scatterplot of KEGG pathway enrichment . (PPTX 215 kb)

Additional file 15: **Figure S10.** KEGG enrichment analysis for genes in “midnightblue” module. **a** Statistic analysis of annotated genes in KEGG pathways. **b** Scatterplot of KEGG pathway enrichment. (PPTX 192 kb)

Additional file 16: **Table S6.** Primers used for qRT-PCR assays. (XLSX 9 kb)

## Abbreviations

AMS: Aborted microspore
COG: Cluster of orthologous goups
DEGs: Differentially expressed genes
DYT1: DYSFUNCIONGAL TAPETUM1
FPKM: Fragments perkilo base of transcript per million mapped reads
GO: Gene ontology
GS: Glutamine synthetase
KEGG: Kyoto encyclopedia of genes and genomes
MS: Male sterility
*orfs*: Open reading frames
qRT-PCR: Quantitative reverse transcription polymerase chain reaction
TDF1: Defective in tapetal development and function1
TF: Transcription factor
WGCNA: Weight correlation network analysis
