# Supplementary material for: The key genes and pathways related to male sterility of eggplant revealed by comparative transcriptome analysis
Source: BMC Plant Biol. 2018 Sep 24;18:209. doi: 10.1186/s12870-018-1430-2 (PMC6154905; doi:10.1186/s12870-018-1430-2)
Supplement: Supplementary file 3 — Figure S1. Analysis of GO enrichment for genes in cluster1. (PPTX 67 kb) [file 12870_2018_1430_MOESM3_ESM.pptx]

## Slide 1
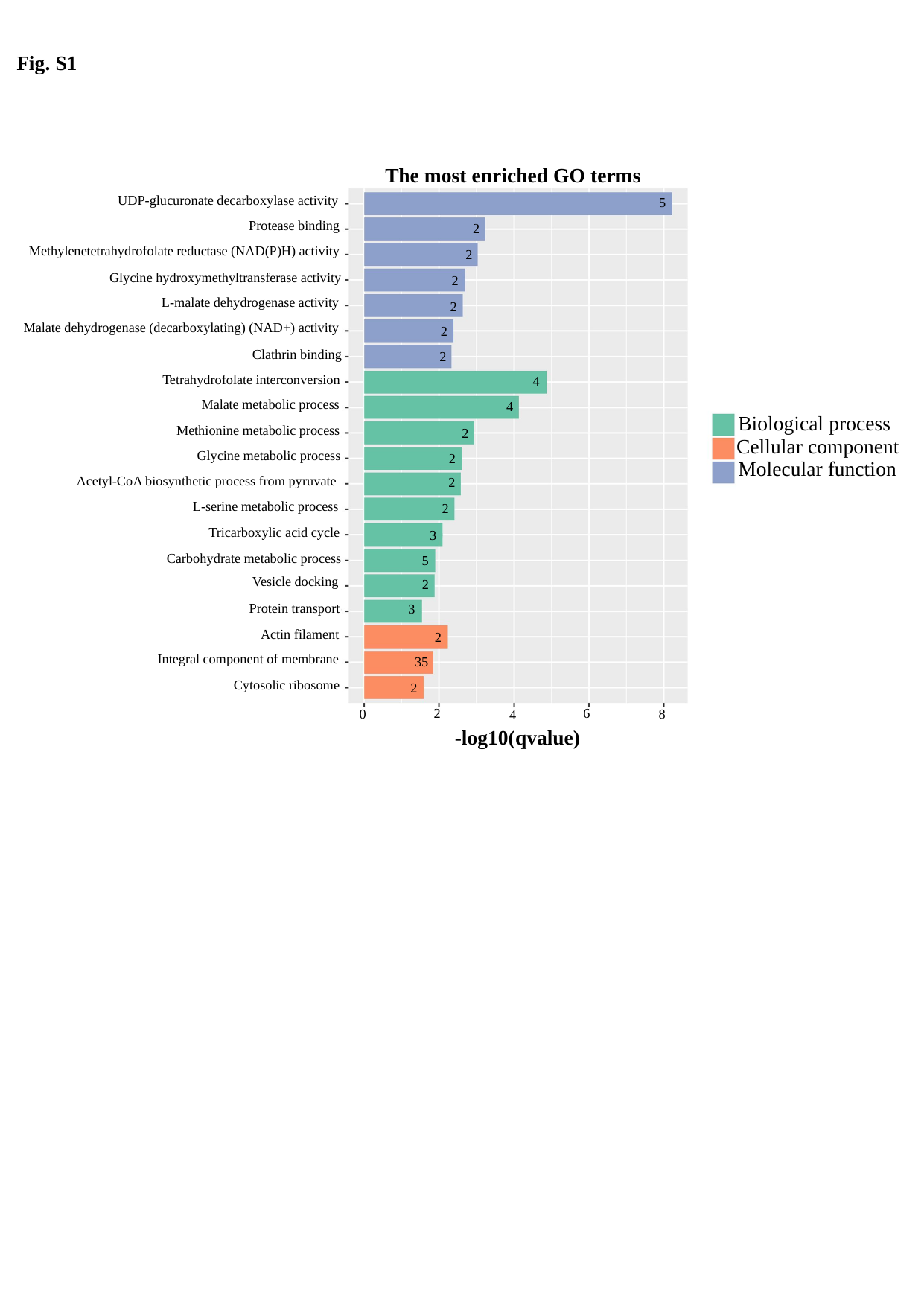

Fig. S1
The most enriched GO terms
UDP-glucuronate decarboxylase activity
5
Protease binding
2
Methylenetetrahydrofolate reductase (NAD(P)H) activity
2
Glycine hydroxymethyltransferase activity
2
L-malate dehydrogenase activity
2
Malate dehydrogenase (decarboxylating) (NAD+) activity
2
Clathrin binding
2
Tetrahydrofolate interconversion
4
Malate metabolic process
4
Biological process
Methionine metabolic process
2
Cellular component
Glycine metabolic process
2
Molecular function
Acetyl-CoA biosynthetic process from pyruvate
2
L-serine metabolic process
2
Tricarboxylic acid cycle
3
Carbohydrate metabolic process
5
Vesicle docking
2
Protein transport
3
Actin filament
2
Integral component of membrane
35
Cytosolic ribosome
2
6
2
8
0
4
-log10(qvalue)
