# Supplementary material for: The key genes and pathways related to male sterility of eggplant revealed by comparative transcriptome analysis
Source: BMC Plant Biol. 2018 Sep 24;18:209. doi: 10.1186/s12870-018-1430-2 (PMC6154905; doi:10.1186/s12870-018-1430-2)
Supplement: Supplementary file 4 — Figure S2. Analysis of GO enrichment for genes in cluster4. (PPTX 66 kb) [file 12870_2018_1430_MOESM4_ESM.pptx]

## Slide 1
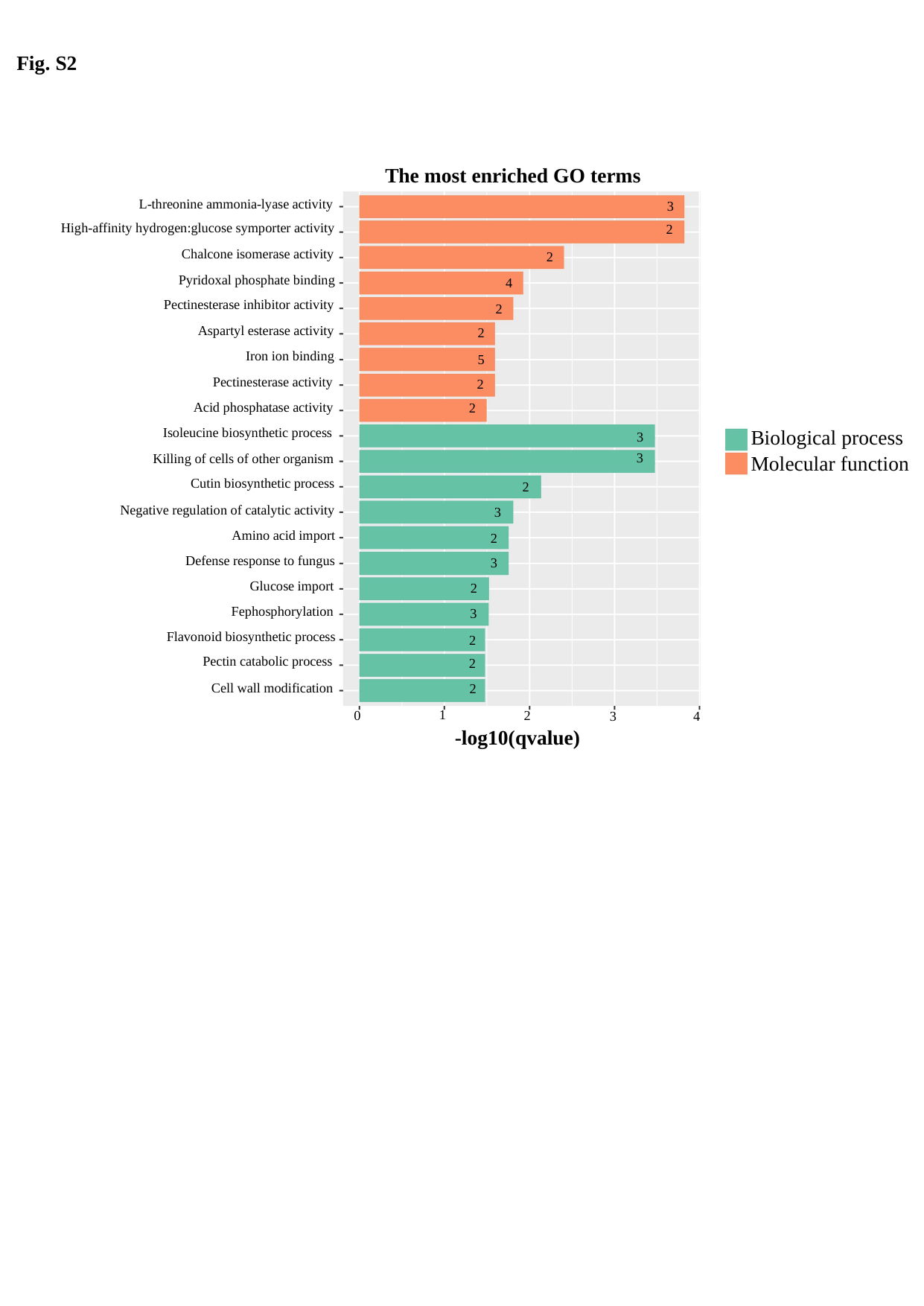

Fig. S2
The most enriched GO terms
L-threonine ammonia-lyase activity
3
High-affinity hydrogen:glucose symporter activity
2
Chalcone isomerase activity
2
Pyridoxal phosphate binding
4
Pectinesterase inhibitor activity
2
Aspartyl esterase activity
2
Iron ion binding
5
Pectinesterase activity
2
Acid phosphatase activity
2
Isoleucine biosynthetic process
Biological process
3
3
Killing of cells of other organism
Molecular function
Cutin biosynthetic process
2
Negative regulation of catalytic activity
3
Amino acid import
2
Defense response to fungus
3
Glucose import
2
Fephosphorylation
3
Flavonoid biosynthetic process
2
Pectin catabolic process
2
Cell wall modification
2
1
0
2
3
4
-log10(qvalue)
