# Supplementary material for: The key genes and pathways related to male sterility of eggplant revealed by comparative transcriptome analysis
Source: BMC Plant Biol. 2018 Sep 24;18:209. doi: 10.1186/s12870-018-1430-2 (PMC6154905; doi:10.1186/s12870-018-1430-2)
Supplement: Supplementary file 5 — Figure S3. Analysis of GO enrichment for genes in cluster5. (PPTX 66 kb) [file 12870_2018_1430_MOESM5_ESM.pptx]

## Slide 1
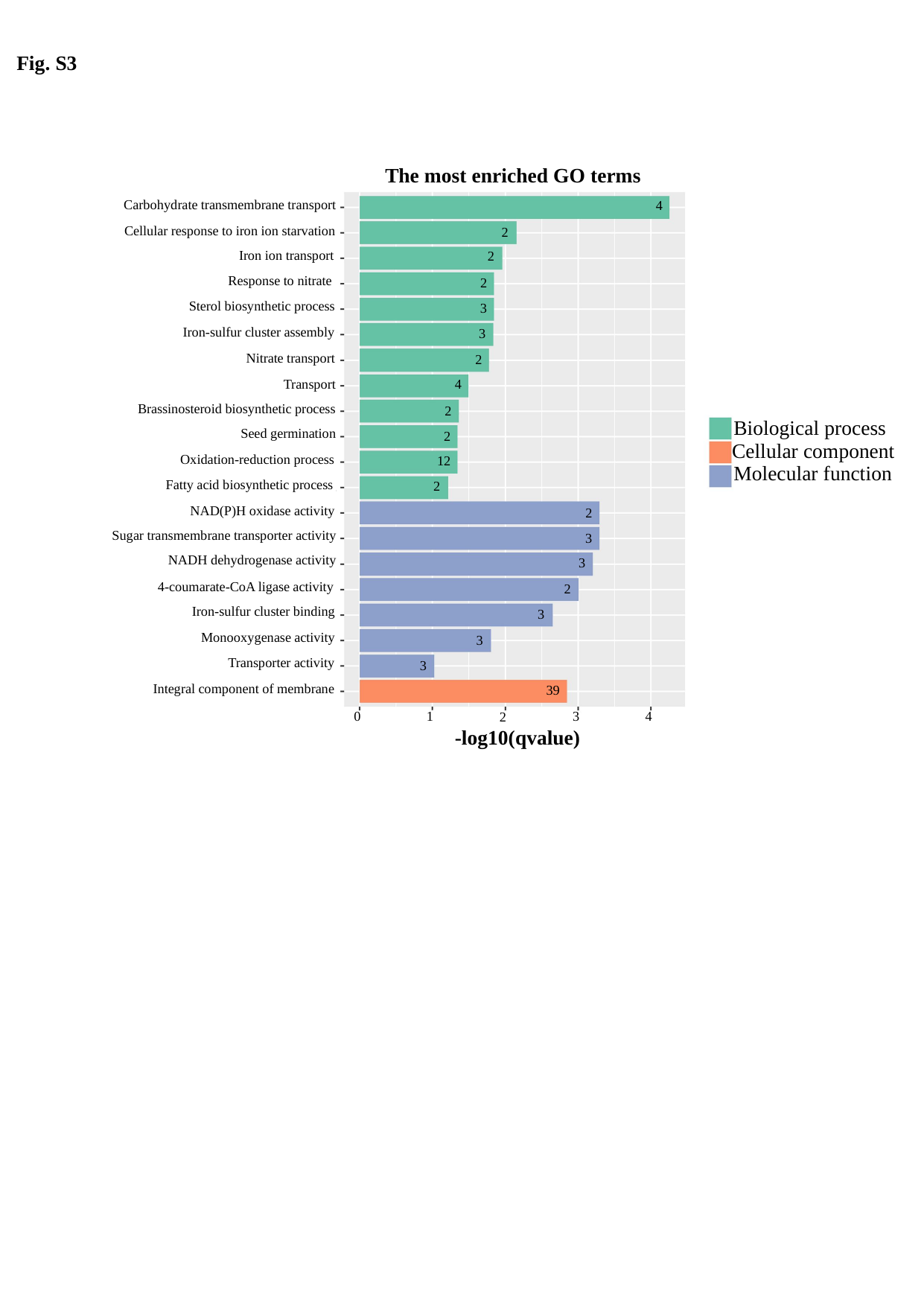

Fig. S3
The most enriched GO terms
Carbohydrate transmembrane transport
4
Cellular response to iron ion starvation
2
Iron ion transport
2
Response to nitrate
2
Sterol biosynthetic process
3
Iron-sulfur cluster assembly
3
Nitrate transport
2
Transport
4
Brassinosteroid biosynthetic process
2
Biological process
Seed germination
2
Cellular component
Oxidation-reduction process
12
Molecular function
Fatty acid biosynthetic process
2
NAD(P)H oxidase activity
2
Sugar transmembrane transporter activity
3
NADH dehydrogenase activity
3
4-coumarate-CoA ligase activity
2
Iron-sulfur cluster binding
3
Monooxygenase activity
3
Transporter activity
3
Integral component of membrane
39
3
1
0
4
2
-log10(qvalue)
