# Supplementary material for: The key genes and pathways related to male sterility of eggplant revealed by comparative transcriptome analysis
Source: BMC Plant Biol. 2018 Sep 24;18:209. doi: 10.1186/s12870-018-1430-2 (PMC6154905; doi:10.1186/s12870-018-1430-2)
Supplement: Supplementary file 6 — Figure S4. Analysis of GO enrichment for genes in cluster3. (PPTX 67 kb) [file 12870_2018_1430_MOESM6_ESM.pptx]

## Slide 1
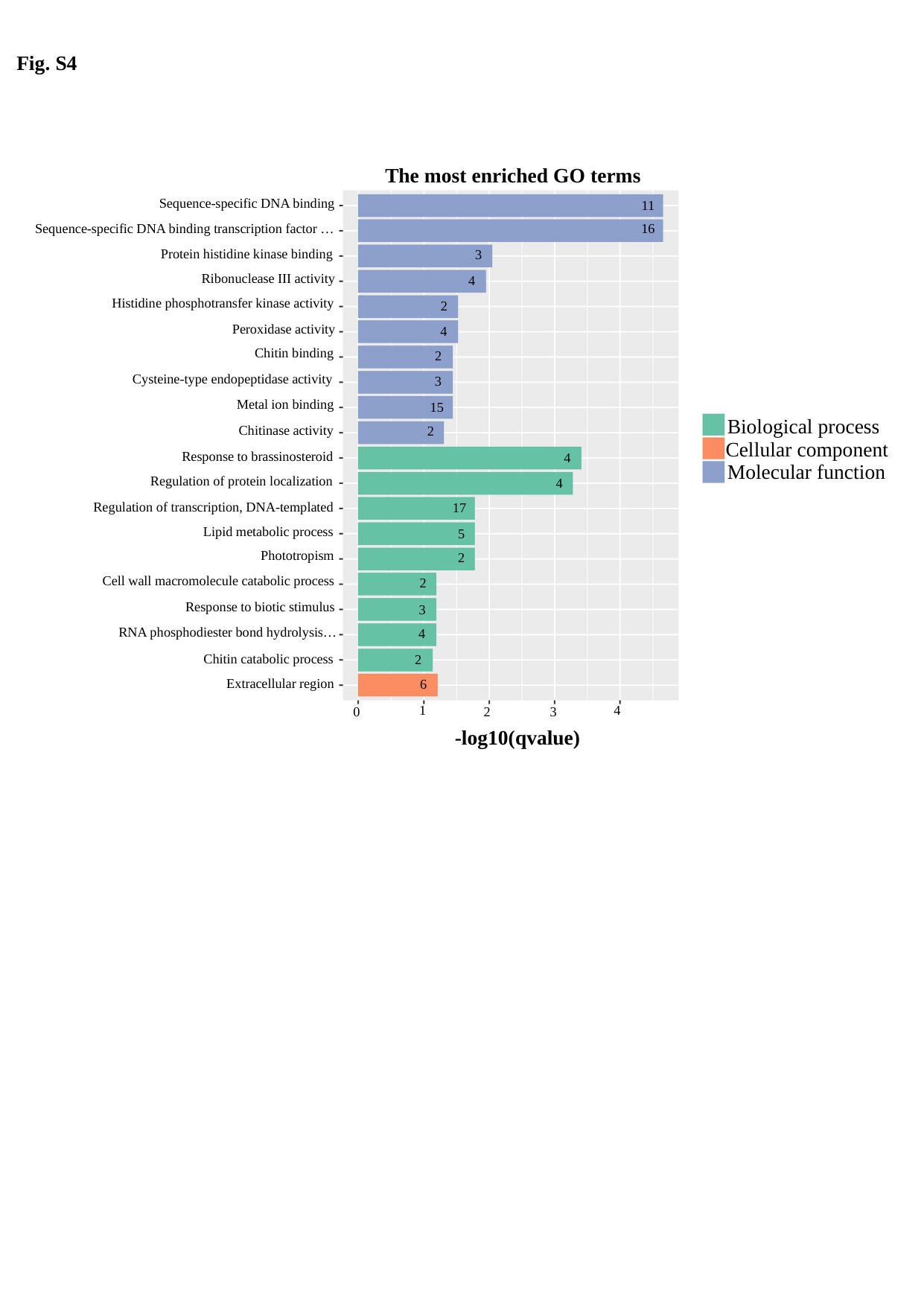

Fig. S4
The most enriched GO terms
Sequence-specific DNA binding
11
16
Sequence-specific DNA binding transcription factor …
Protein histidine kinase binding
3
Ribonuclease III activity
4
Histidine phosphotransfer kinase activity
2
Peroxidase activity
4
Chitin binding
2
Cysteine-type endopeptidase activity
3
Metal ion binding
15
Biological process
Chitinase activity
2
Cellular component
Response to brassinosteroid
4
Molecular function
Regulation of protein localization
4
Regulation of transcription, DNA-templated
17
Lipid metabolic process
5
Phototropism
2
Cell wall macromolecule catabolic process
2
Response to biotic stimulus
3
RNA phosphodiester bond hydrolysis…
4
Chitin catabolic process
2
Extracellular region
6
1
4
0
3
2
-log10(qvalue)
