# Supplementary material for: The key genes and pathways related to male sterility of eggplant revealed by comparative transcriptome analysis
Source: BMC Plant Biol. 2018 Sep 24;18:209. doi: 10.1186/s12870-018-1430-2 (PMC6154905; doi:10.1186/s12870-018-1430-2)
Supplement: Supplementary file 7 — Figure S5. Analysis of GO enrichment for genes in cluster7. (PPTX 66 kb) [file 12870_2018_1430_MOESM7_ESM.pptx]

## Slide 1
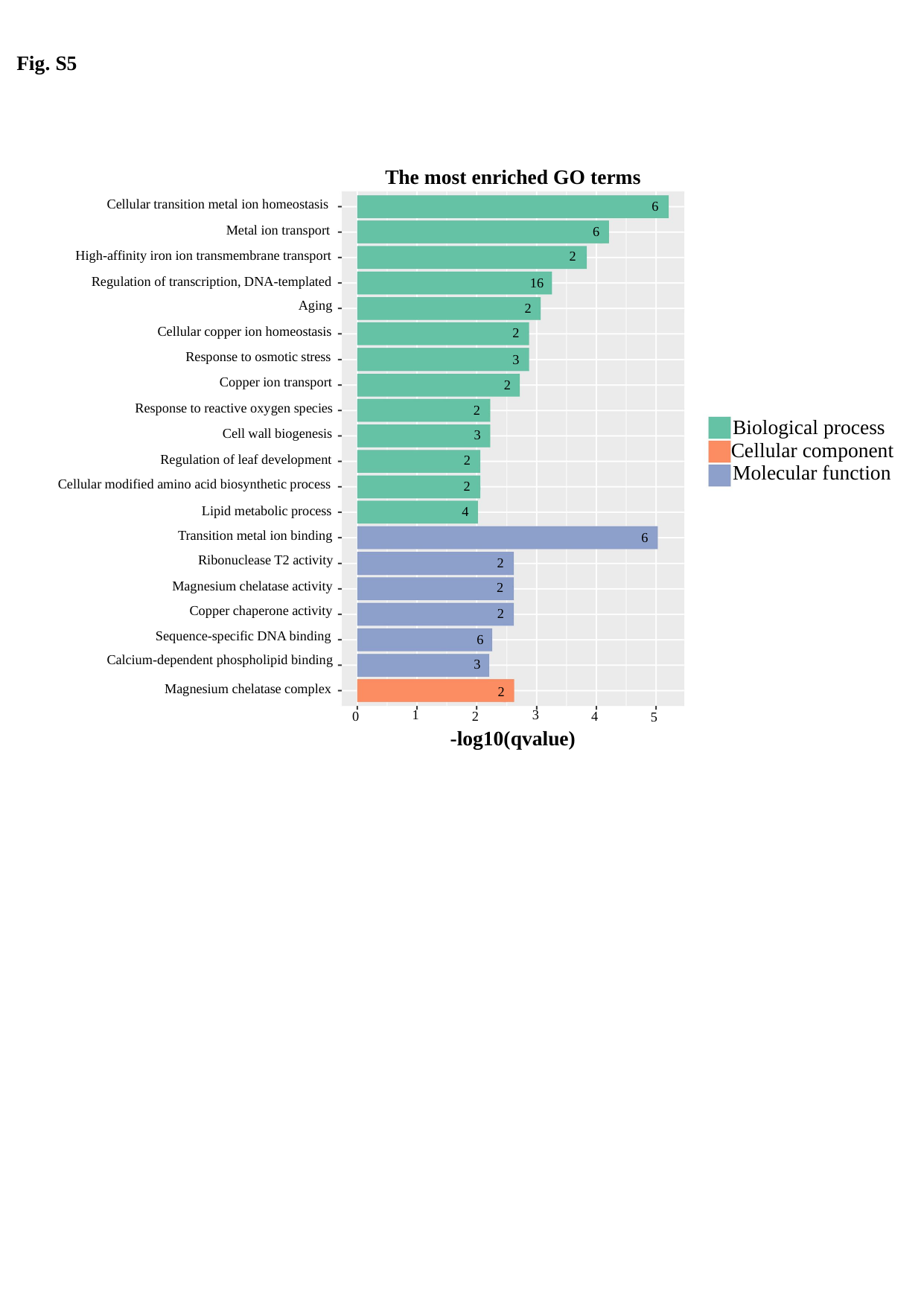

Fig. S5
The most enriched GO terms
Cellular transition metal ion homeostasis
6
Metal ion transport
6
High-affinity iron ion transmembrane transport
2
Regulation of transcription, DNA-templated
16
Aging
2
Cellular copper ion homeostasis
2
Response to osmotic stress
3
Copper ion transport
2
Response to reactive oxygen species
2
Biological process
Cell wall biogenesis
3
Cellular component
Regulation of leaf development
2
Molecular function
Cellular modified amino acid biosynthetic process
2
Lipid metabolic process
4
Transition metal ion binding
6
Ribonuclease T2 activity
2
Magnesium chelatase activity
2
Copper chaperone activity
2
Sequence-specific DNA binding
6
Calcium-dependent phospholipid binding
3
Magnesium chelatase complex
2
1
3
2
4
0
5
-log10(qvalue)
