# Supplementary material for: The key genes and pathways related to male sterility of eggplant revealed by comparative transcriptome analysis
Source: BMC Plant Biol. 2018 Sep 24;18:209. doi: 10.1186/s12870-018-1430-2 (PMC6154905; doi:10.1186/s12870-018-1430-2)
Supplement: Supplementary file 8 — Figure S6. Analysis of GO enrichment for genes in cluster9. (PPTX 64 kb) [file 12870_2018_1430_MOESM8_ESM.pptx]

## Slide 1
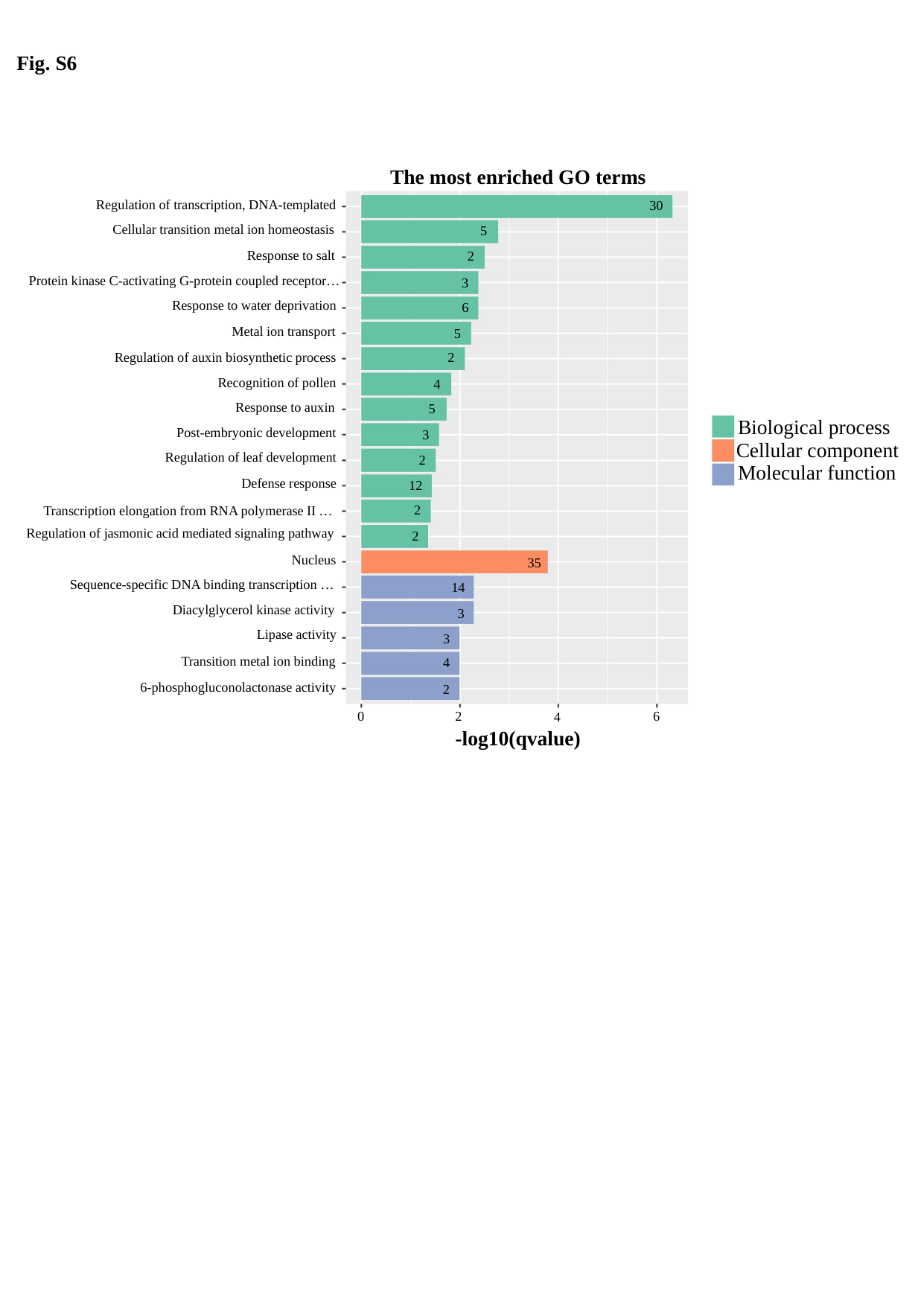

Fig. S6
The most enriched GO terms
Regulation of transcription, DNA-templated
30
Cellular transition metal ion homeostasis
5
Response to salt
2
Protein kinase C-activating G-protein coupled receptor…
3
Response to water deprivation
6
Metal ion transport
5
Regulation of auxin biosynthetic process
2
Recognition of pollen
4
Response to auxin
5
Biological process
Post-embryonic development
3
Cellular component
Regulation of leaf development
2
Molecular function
Defense response
12
2
Transcription elongation from RNA polymerase II …
Regulation of jasmonic acid mediated signaling pathway
2
Nucleus
35
Sequence-specific DNA binding transcription …
14
Diacylglycerol kinase activity
3
Lipase activity
3
Transition metal ion binding
4
6-phosphogluconolactonase activity
2
2
0
6
4
-log10(qvalue)
