# Supplementary material for: The key genes and pathways related to male sterility of eggplant revealed by comparative transcriptome analysis
Source: BMC Plant Biol. 2018 Sep 24;18:209. doi: 10.1186/s12870-018-1430-2 (PMC6154905; doi:10.1186/s12870-018-1430-2)
Supplement: Supplementary file 12 — Figure S7. Analysis of GO enrichment for genes in “lightcyan” module. (PPTX 128 kb) [file 12870_2018_1430_MOESM12_ESM.pptx]

## Slide 1
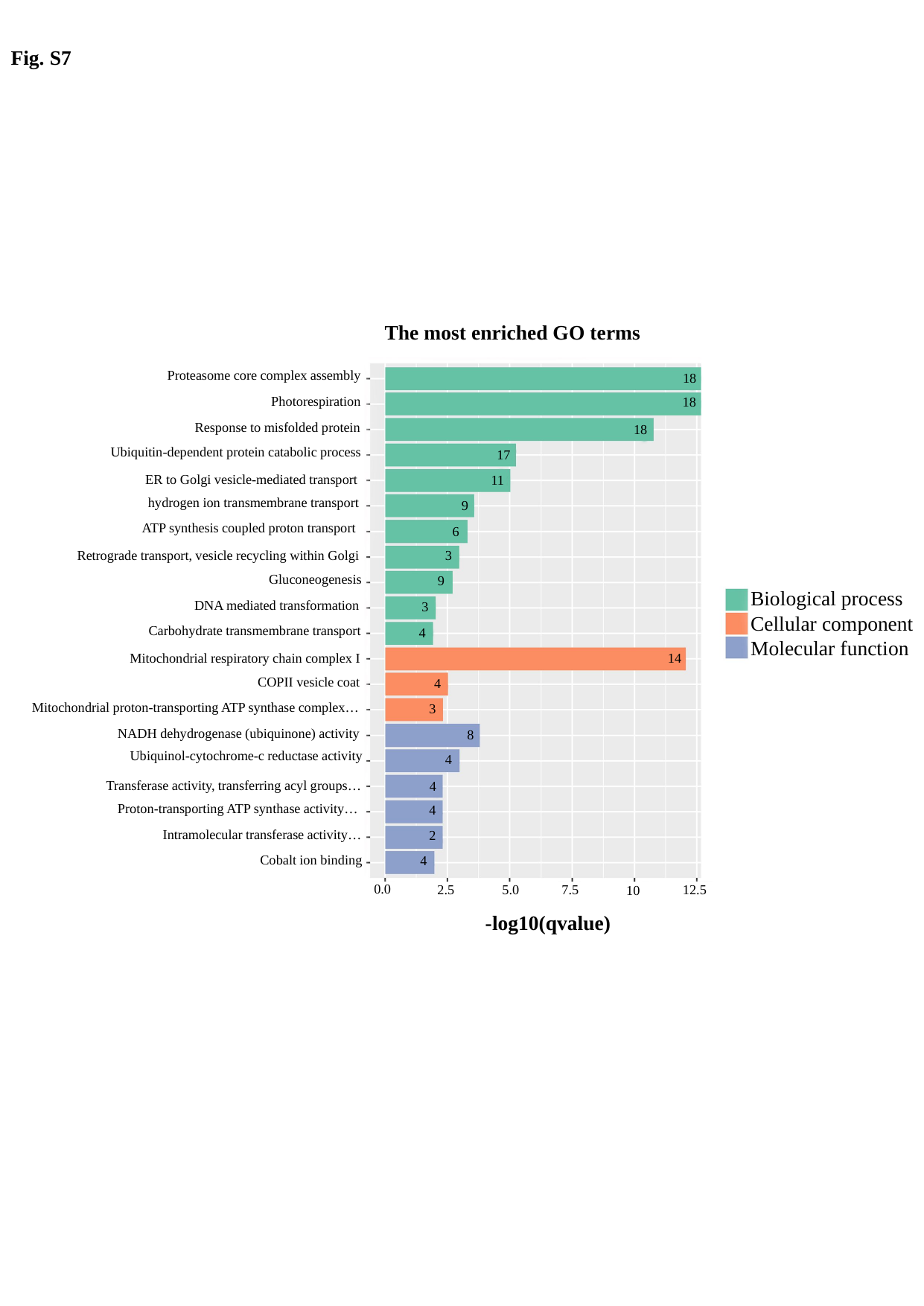

Fig. S7
The most enriched GO terms
Proteasome core complex assembly
18
Photorespiration
18
Response to misfolded protein
18
Ubiquitin-dependent protein catabolic process
17
ER to Golgi vesicle-mediated transport
11
hydrogen ion transmembrane transport
9
ATP synthesis coupled proton transport
6
Retrograde transport, vesicle recycling within Golgi
3
Gluconeogenesis
9
Biological process
DNA mediated transformation
3
Cellular component
Carbohydrate transmembrane transport
4
Molecular function
Mitochondrial respiratory chain complex I
14
COPII vesicle coat
4
Mitochondrial proton-transporting ATP synthase complex…
3
NADH dehydrogenase (ubiquinone) activity
8
Ubiquinol-cytochrome-c reductase activity
4
Transferase activity, transferring acyl groups…
4
Proton-transporting ATP synthase activity…
4
Intramolecular transferase activity…
2
Cobalt ion binding
4
0.0
5.0
7.5
12.5
2.5
10
-log10(qvalue)
