# Supplementary material for: The key genes and pathways related to male sterility of eggplant revealed by comparative transcriptome analysis
Source: BMC Plant Biol. 2018 Sep 24;18:209. doi: 10.1186/s12870-018-1430-2 (PMC6154905; doi:10.1186/s12870-018-1430-2)
Supplement: Supplementary file 13 — Figure S8. Analysis of GO enrichment for genes in “midnightblue” module. (PPTX 83 kb) [file 12870_2018_1430_MOESM13_ESM.pptx]

## Slide 1
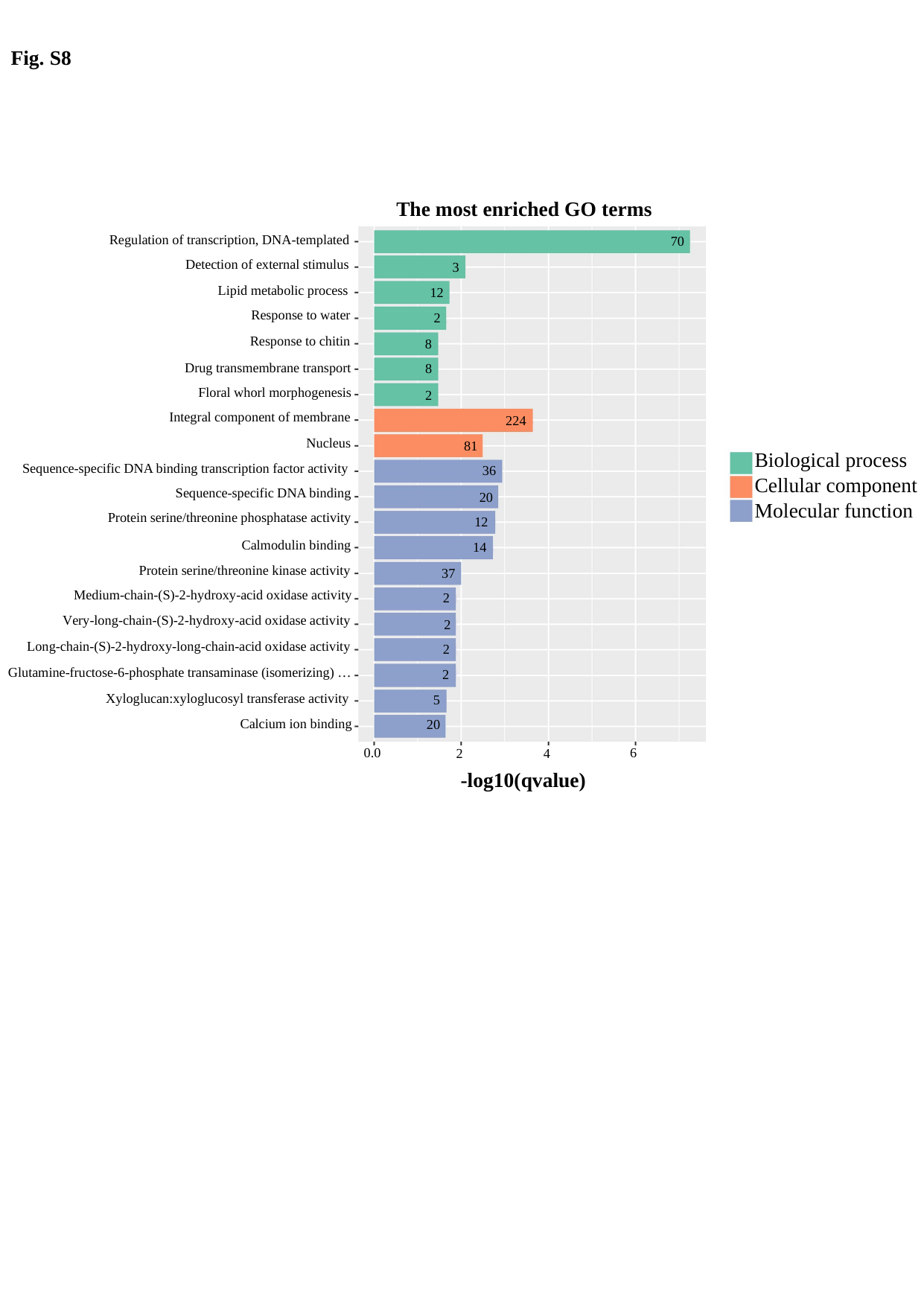

Fig. S8
The most enriched GO terms
Regulation of transcription, DNA-templated
70
Detection of external stimulus
3
Lipid metabolic process
12
Response to water
2
Response to chitin
8
Drug transmembrane transport
8
Floral whorl morphogenesis
2
Integral component of membrane
224
Nucleus
81
Biological process
Sequence-specific DNA binding transcription factor activity
36
Cellular component
Sequence-specific DNA binding
20
Molecular function
Protein serine/threonine phosphatase activity
12
Calmodulin binding
14
Protein serine/threonine kinase activity
37
Medium-chain-(S)-2-hydroxy-acid oxidase activity
2
Very-long-chain-(S)-2-hydroxy-acid oxidase activity
2
Long-chain-(S)-2-hydroxy-long-chain-acid oxidase activity
2
Glutamine-fructose-6-phosphate transaminase (isomerizing) …
2
Xyloglucan:xyloglucosyl transferase activity
5
Calcium ion binding
20
0.0
6
4
2
-log10(qvalue)
