# Supplementary material for: The key genes and pathways related to male sterility of eggplant revealed by comparative transcriptome analysis
Source: BMC Plant Biol. 2018 Sep 24;18:209. doi: 10.1186/s12870-018-1430-2 (PMC6154905; doi:10.1186/s12870-018-1430-2)
Supplement: Supplementary file 14 — Figure S9. KEGG enrichment analysis for genes in “lightcyan” module. a Statistic analysis of annotated genes in KEGG pathways. b Scatterplot of KEGG pathway enrichment . (PPTX 215 kb) [file 12870_2018_1430_MOESM14_ESM.pptx]

## Slide 1
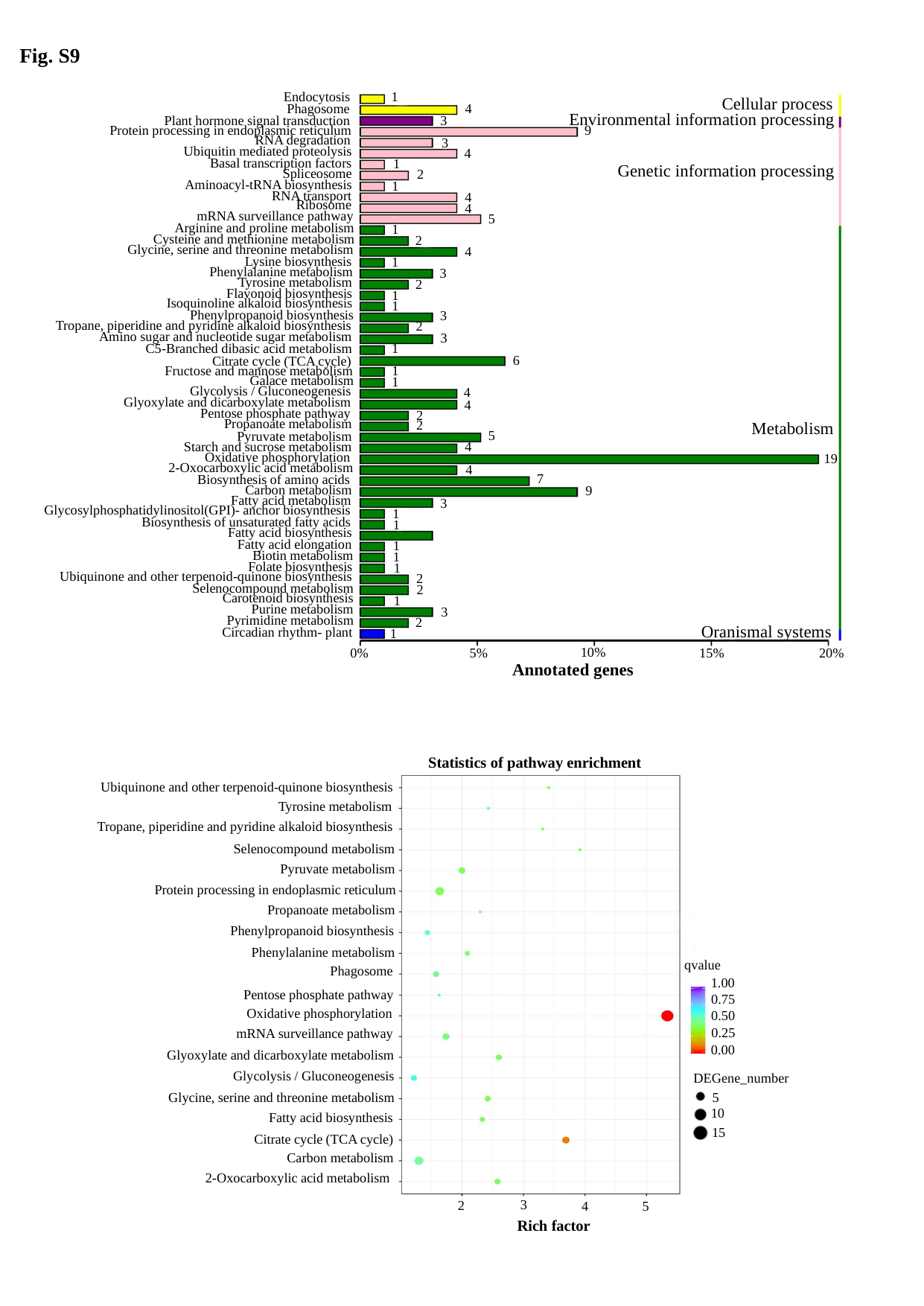

Fig. S9
Endocytosis
1
4
Phagosome
Plant hormone signal transduction
3
Protein processing in endoplasmic reticulum
9
RNA degradation
3
Ubiquitin mediated proteolysis
4
Basal transcription factors
1
Spliceosome
2
Aminoacyl-tRNA biosynthesis
1
RNA transport
4
Ribosome
4
mRNA surveillance pathway
5
Arginine and proline metabolism
1
Cysteine and methionine metabolism
2
Glycine, serine and threonine metabolism
4
Lysine biosynthesis
1
Phenylalanine metabolism
3
Tyrosine metabolism
2
Flavonoid biosynthesis
1
Isoquinoline alkaloid biosynthesis
1
Phenylpropanoid biosynthesis
3
2
Amino sugar and nucleotide sugar metabolism
3
C5-Branched dibasic acid metabolism
1
6
Citrate cycle (TCA cycle)
1
Fructose and mannose metabolism
Galace metabolism
1
Glycolysis / Gluconeogenesis
4
Glyoxylate and dicarboxylate metabolism
4
Pentose phosphate pathway
2
Propanoate metabolism
2
5
Pyruvate metabolism
Starch and sucrose metabolism
4
Oxidative phosphorylation
2-Oxocarboxylic acid metabolism
4
7
Biosynthesis of amino acids
Carbon metabolism
9
Fatty acid metabolism
3
1
Biosynthesis of unsaturated fatty acids
1
Fatty acid biosynthesis
Fatty acid elongation
1
Biotin metabolism
1
Folate biosynthesis
1
2
Selenocompound metabolism
2
Carotenoid biosynthesis
1
Purine metabolism
3
Pyrimidine metabolism
2
Circadian rhythm- plant
1
5%
0%
Genetic information processing
Metabolism
19
Oranismal systems
10%
20%
Annotated genes
Cellular process
Environmental information processing
Tropane, piperidine and pyridine alkaloid biosynthesis
Glycosylphosphatidylinositol(GPI)- anchor biosynthesis
Ubiquinone and other terpenoid-quinone biosynthesis
15%
Statistics of pathway enrichment
Ubiquinone and other terpenoid-quinone biosynthesis
Tyrosine metabolism
Tropane, piperidine and pyridine alkaloid biosynthesis
Selenocompound metabolism
Pyruvate metabolism
Protein processing in endoplasmic reticulum
Propanoate metabolism
Phenylpropanoid biosynthesis
Phenylalanine metabolism
qvalue
Phagosome
1.00
Pentose phosphate pathway
0.75
Oxidative phosphorylation
0.50
0.25
mRNA surveillance pathway
0.00
Glyoxylate and dicarboxylate metabolism
Glycolysis / Gluconeogenesis
DEGene_number
Glycine, serine and threonine metabolism
5
10
Fatty acid biosynthesis
15
Citrate cycle (TCA cycle)
Carbon metabolism
2-Oxocarboxylic acid metabolism
3
2
4
5
Rich factor
