# Supplementary material for: The key genes and pathways related to male sterility of eggplant revealed by comparative transcriptome analysis
Source: BMC Plant Biol. 2018 Sep 24;18:209. doi: 10.1186/s12870-018-1430-2 (PMC6154905; doi:10.1186/s12870-018-1430-2)
Supplement: Supplementary file 15 — Figure S10. KEGG enrichment analysis for genes in “midnightblue” module. a Statistic analysis of annotated genes in KEGG pathways. b Scatterplot of KEGG pathway enrichment. (PPTX 192 kb) [file 12870_2018_1430_MOESM15_ESM.pptx]

## Slide 1
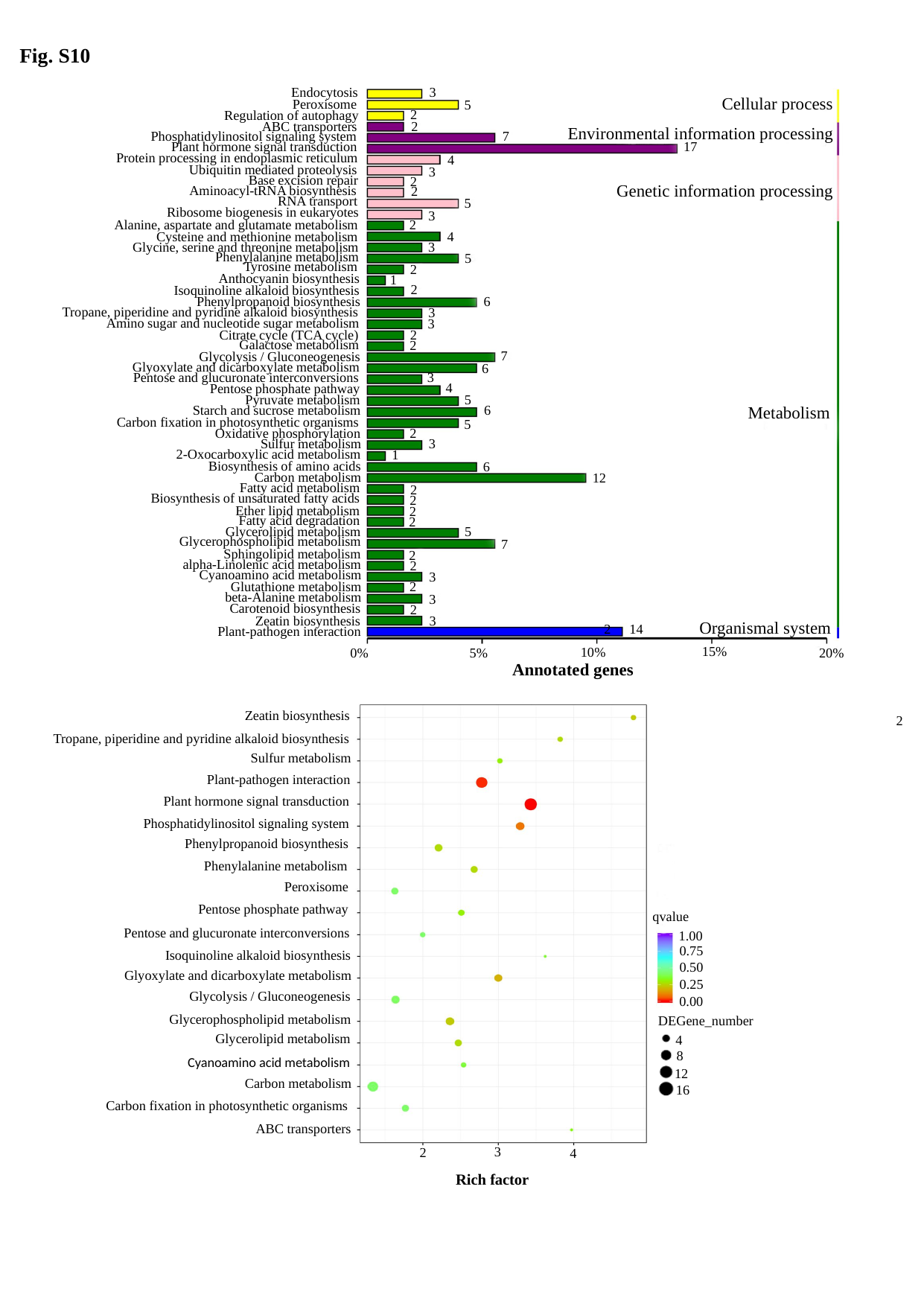

Fig. S10
3
Endocytosis
Peroxisome
Regulation of autophagy
ABC transporters
Phosphatidylinositol signaling system
Protein processing in endoplasmic reticulum
Base excision repair
RNA transport
Alanine, aspartate and glutamate metabolism
Glycine, serine and threonine metabolism
Phenylalanine metabolism
Tyrosine metabolism
Amino sugar and nucleotide sugar metabolism
Citrate cycle (TCA cycle)
Galactose metabolism
Glycolysis / Gluconeogenesis
Glyoxylate and dicarboxylate metabolism
Pentose and glucuronate interconversions
Carbon fixation in photosynthetic organisms
2-Oxocarboxylic acid metabolism
Biosynthesis of amino acids
Carbon metabolism
Fatty acid metabolism
Biosynthesis of unsaturated fatty acids
Ether lipid metabolism
Fatty acid degradation
Glycerophospholipid metabolism
Sphingolipid metabolism
alpha-Linolenic acid metabolism
Cyanoamino acid metabolism
Glutathione metabolism
beta-Alanine metabolism
Plant-pathogen interaction
Cellular process
5
2
2
Environmental information processing
7
Plant hormone signal transduction
17
4
Ubiquitin mediated proteolysis
3
2
Genetic information processing
Aminoacyl-tRNA biosynthesis
2
5
Ribosome biogenesis in eukaryotes
3
2
Cysteine and methionine metabolism
4
3
5
2
Anthocyanin biosynthesis
1
2
Isoquinoline alkaloid biosynthesis
6
Phenylpropanoid biosynthesis
Tropane, piperidine and pyridine alkaloid biosynthesis
3
3
2
2
7
6
3
4
Pentose phosphate pathway
Pyruvate metabolism
5
6
Starch and sucrose metabolism
Metabolism
5
2
Oxidative phosphorylation
3
Sulfur metabolism
1
6
12
2
2
2
2
5
Glycerolipid metabolism
7
2
2
3
2
3
Carotenoid biosynthesis
2
3
Zeatin biosynthesis
Organismal system
2
14
15%
10%
5%
20%
0%
Annotated genes
Zeatin biosynthesis
2
Tropane, piperidine and pyridine alkaloid biosynthesis
Sulfur metabolism
Plant-pathogen interaction
Plant hormone signal transduction
Phosphatidylinositol signaling system
Phenylpropanoid biosynthesis
Phenylalanine metabolism
Peroxisome
Pentose phosphate pathway
qvalue
Pentose and glucuronate interconversions
1.00
0.75
Isoquinoline alkaloid biosynthesis
0.50
Glyoxylate and dicarboxylate metabolism
0.25
Glycolysis / Gluconeogenesis
0.00
Glycerophospholipid metabolism
DEGene_number
Glycerolipid metabolism
4
8
Cyanoamino acid metabolism
12
Carbon metabolism
16
Carbon fixation in photosynthetic organisms
ABC transporters
3
2
4
Rich factor
